# A cross-sectional evaluation of community pharmacists’ opinions on caring for visually impaired patients: pharmacist experiences, confidence and recommendations for improved care

**DOI:** 10.1007/s11096-026-02120-4

**Published:** 2026-03-18

**Authors:** Aoife Goodwin-Boers, Harriet Bennett-Lenane

**Affiliations:** School of Pharmacy, University College Cork, Pharmacy Building, College Road, Cork, Ireland

**Keywords:** Care, Community pharmacy service, Patient, Persons, Visually impaired

## Abstract

**Introduction:**

Health inequalities and medication management challenges are apparent for people with disabilities, including those who are visually impaired (VI). Community pharmacists are well placed to provide advice and support to VI patients. It is imperative for appropriate care that they are adequately prepared to adapt practice for these patients.

**Aim:**

The study aimed to elucidate current experiences, perceived confidence and priorities of community pharmacists when caring for VI patients. It also aimed to understand current barriers and recommendations for practice adaptations to improve care of this cohort.

**Method:**

This study utilised an online cross-sectional questionnaire comprising multiple choice, Likert scale and open text questions. This questionnaire was disseminated to registered community pharmacists in Ireland. Responses were coded and analysed quantitatively using descriptive and inferential statistics—whereby *p* < 0.05 denotes statistical significance. Open-text responses were analysed using conventional content analysis.

**Results:**

Data were collected from 235 pharmacists (5.4% response rate). Approximately 70% (69.8%, n = 164) had previous experience providing care to VI patients, while over 90% (91.9%, n = 214) believed that VI patients are at increased risk of medication related harm. Pharmacists with over 10 years’ experience (75.4% n = 98) were significantly more likely to feel confident caring for VI patients than those less experienced (52.9%, n = 18), *p* < 0.05. Only 10.3% (n = 24) believed the pharmacy teams they work with were adequately trained to care for VI patients, while 69.0% (n = 162) do not believe they have been provided with sufficient training and guidance on this topic. Most pharmacists would welcome training to identify (90.6%, n = 213) and guide management of VI patients (92.3%, n = 216). Open text responses revealed recommendations of adaptions to care. Respondent recommendations related to pharmacy physical layouts, patient identification, tailored medication dispensing practices, use of technology and improved communication methods.

**Conclusion:**

This study revealed valuable insights into Irish community pharmacists’ experiences, confidence and recommendations for adapting care for VI patients. Current barriers in addition to confidence and knowledge deficits and a lack of relevant training amongst pharmacists were identified. Further emphasis on this topic in undergraduate pharmacy programmes and continuing professional development training can help community pharmacists provide equitable care to VI patients.

**Supplementary Information:**

The online version contains supplementary material available at 10.1007/s11096-026-02120-4.

## Impact statements

- At present community pharmacists observe a lack of support in how to adapt their practice for visually impaired patients, owing to a deficiency in training and guidance dissemination.
- Community pharmacist recommendations include tailored dispensing practices, use of technology and improved communication methods.
- Creativity is required in the methods of delivery of training and awareness campaigns to ensure accessibility of pharmacists and students to relevant information.

## Introduction

Presence of a disability can often lead to poorer health outcomes for individuals [1]. Patient cohorts, such as those who are visually impaired (VI), often face challenges regarding access to healthcare, leading to challenging or negative healthcare experiences [2]. This often results in disparities in health-related quality of life and functioning versus sighted individuals [3]. This inequity poses a significant challenge for modern healthcare systems internationally. Recent estimates suggest over 2.2 billion people worldwide are VI [4]. In particular, incidences of acquired VI (i.e. develops after birth due to disease, injury or trauma) which accounts for approximately 90% of cases, are increasing year on year due to a rapidly aging worldwide population [3, 5].

Previous research has highlighted substantial medication management difficulties for VI patients [6–11]. This cohort can struggle with tasks related to medication information access, along with medication identification and administration. As VI adults are more likely to require daily medication [12], this sensory impairment poses a significant issue for medication adherence and independent medication use. Additionally, it was previously reported that older people with VI are more than twice as likely to need help in managing medication [13]. VI has a significant impact on medication safety and individualised or tailored management approaches are required to support these patients.

Community pharmacists are accessible healthcare professionals and medicines experts at the heart of the community. They are well placed to support VI patients in medication management and improve their access to equitable healthcare delivery. Previous international studies have highlighted varied experiences of VI patients on the quality of care provided in community pharmacies [5, 14–19]. Various barriers and facilitators to care have been established. For example, the importance of and preference for attending a regular community pharmacy by VI patients has been identified [14]. However, despite evidence of excellent patient-pharmacist relationships [15], inappropriate communication and counselling regarding medications [5, 15, 17], lack of disability awareness and poor pharmacy layouts were previously highlighted as barriers to care by VI patients [14, 15, 18, 19]. These factors have led to uncomfortable interactions with community pharmacy staff [16], coupled with a perceived lack of independence versus the sighted population [14].

The experiences and perceptions of community pharmacists on providing care to VI patients is relatively underexplored in literature to date. Previously published studies have primarily involved qualitative explorations of pharmacist’s views in countries such as Belgium, Saudi Arabia, Scotland and Thailand [18, 20–22]. Given the substantial role community pharmacists can play in improving medication management for VI patients, further data are required on pharmacist confidence and perceived readiness to care for this cohort. Furthermore, obtaining recommendations for practice adaptions and understanding current barriers to care from practicing community pharmacists could aid in development of improved guidance for pharmacy staff or inform educational material for undergraduate students.

### Aim

The study aimed to elucidate the current experiences, perceived confidence and priorities of community pharmacists when caring for VI patients. An understanding of current barriers to care and recommendations for practice adaptations to improve care of these patients were also explored.

## Method

### Study design and participants

Participants eligible for inclusion were pharmacists registered with the Pharmaceutical Society of Ireland (PSI) who work in the community pharmacy sector (either part-time or full-time). A survey study design was chosen to facilitate widespread data collection. The Strengthening the Reporting of Observational Studies in Epidemiology (STROBE) Statement was used to guide study reporting [23].

### Questionnaire design and distribution

The questionnaire (Supplementary Information 1) was developed based on a literature review of existing related research [18, 20–22]. The questionnaire was created on Microsoft Forms (Microsoft Office 2016) and consisted of a combination of Likert scale ratings, multiple choice, and open text questions. The questionnaire was piloted with three community pharmacists to ensure face validity and content validity. Their data were not included in the final analysis. An information sheet preceding the questionnaire highlighted that any identifying information would be removed prior to data analysis and provided a definition of vision impairment (i.e. All levels of sight loss, covering moderate sight loss, severe sight loss and blindness). A list was obtained from the PSI containing email addresses of all registered pharmacists who list community pharmacy as their area of practice (n = 4,366). An email containing a link to the questionnaire was sent to these pharmacists in October 2024 asking them to participate. Efforts to maximise response rate included a reminder email sent two weeks later, and emails sent to professional contacts of the research team.

### Data analysis

A valid response was achieved when a participant clicked ‘submit’ at the end of the questionnaire. Data from the questionnaire were cross-checked to remove identifying information, subsequently coded and thereafter analysed using descriptive and inferential statistics using Statistical Package for Social Sciences (SPSS), Version 27. Chi-squared (χ2) tests were conducted to examine the association between pharmacist demographics and their responses. Post-hoc analysis was conducted using the z-test to compare column proportions; adjustment for multiple testing using the Bonferroni method was performed. Responses from the open text questions were analysed using conventional content analysis using the steps outlined by Hseish and Shannon [24] using NVivo® (Version 15). Discussion amongst authors occurred at all stages of conventional content analysis to improve data credibility and confirmability. Reflexivity was considered throughout the study to minimise the potential for researcher bias to influence the study findings. At the time of the study AGB (female) was a 5th year pharmacy student who had undertaken a literature review on pharmacist interventions to support pharmaceutical care of patients with visual impairment. HBL (female) was a practising community pharmacist and academic who had previously conducted qualitative research with visually impaired patients. Neither researcher was visually impaired. Consideration of how experiences of the authors before and after the study may have influenced the research was undertaken and the authors ensured that interpretations were grounded in the data as best as possible.

### Ethics approval

The School of Pharmacy Social Research Ethics Committee (SREC) of University College Cork provided approval (Ref: 2024-018).

## Results

### Respondent demographics

A response rate of 5.4% (n = 235) was achieved. Respondent demographics can be seen in Table 1, where almost two thirds (63.8%, n = 150) were female, which correlates closely to the latest PSI figures for registered pharmacists in Ireland, where as of February 2026 64.38% are female. Over three quarters (76.2%, n = 179) had greater than 10 years’ experience working in community pharmacy. Close to half of respondents worked in independent pharmacies (49.4%, n = 116) while there was a relatively even split of employment locations between urban and rural areas.

**Table 1:** Respondent demographics

Demographic	Overall % (n)	Superintendent pharmacist % (n)	Supervising pharmacist % (n)	Support pharmacist % (n)	Locum pharmacist % (n)	Pharmacy owner % (n)	Other % (n)
Gender							
Male	35.3 (83)	8.4% (7)	20.5% (17)	24.1% (20)	13.3% (11)	33.7% (28)	0.0% (0)
Female	63.8% (150)	5.3% (8)	25.3% (38)	32% (48)	24.7% (37)	10% (15)	2.7% (4)
Prefer not to answer	0.9% (2)	0.0% (0)	50% (1)	50% (1)	0.0% (0)	0.0% (0)	0.0% (0)
Years of post registration experience							
≤ 10 years	23.8% (56)	0.0% (0)	26.8% (15)	41.1% (23)	30.4% (17)	0.0% (0)	1.8% (1)
> 10 years	76.2% (179)	8.4% (15)	22.9% (41)	25.7% (46)	17.3% (31)	24.0% (43)	1.7% (3)
Pharmacy type							
Large pharmacy chain ≥ 10 pharmacies	20.9% (49)	0.0% 0%	30.6% (15)	36.7% (18)	28.6% (14)	2.0% (1)	2.0% (1)
Small pharmacy chain < 10 pharmacies	29.8% (70)	5.7% (4)	30.0% (21)	31.4% (22)	20.0% (14)	12.9% (9)	0.0% (0)
Independent pharmacy	49.3% (116)	9.5% (11)	17.2% (20)	25% (29)	17.2% (20)	28.4% (33)	2.6% (3)
Pharmacy location							
Large town	27.4% (64)	3.1% (2)	32.8% (21)	29.7% (19)	15.6% (10)	18.8) (12)	0.0% (0)
City centre or surrounds	31.6% (74)	12.2% (9)	18.9% (14)	32.4% (24)	23.0% (17)	10.8% (8)	2.7% (2)
Rural village or small town	41% (96)	4.2% (4)	20.8% (20)	27.1% (26)	21.9% (21)	24.0% (23)	2.1% (2)

### Current experiences, perceived confidence and level of preparedness to care for VI patients

Almost 70% of pharmacists had prior experience providing care or advice to VI patients (69.8% n = 164) and 91.9% (n = 214) believed that this cohort are at increased risk of medication harm. However, 78.1% (n = 128) of pharmacists with previous experience providing care had never asked for feedback from VI patients. Most respondents (94.0%, n = 221) have not completed any training (formal or informal) regarding caring for VI patients and almost two-thirds (n = 147, 62.6%) do not know how to obtain accessible formats (e.g. braille or audio) of medicine-related information. Over two thirds, 67.9% (n = 159), believed their current pharmacy layout was accessible to VI patients. However, a significantly smaller proportion of pharmacists who worked in a large chain pharmacy (42.9%, n = 21) believed their pharmacy was accessible, compared to those who worked in small chain (71.4%, n = 50) or independent (76.5%, n = 88) pharmacies (*p* < 0.05).

Pharmacists with greater than ten years pharmacy experience were significantly more likely to report feeling confident in providing care (75.4%, n = 98) versus respondents with ≤ 10 years’ experience (52.9% n = 18); *p* < 0.05). Regarding preparedness, as seen in Table 2, responses varied where 31.2% (n = 73) agreed and 30.3% (n = 71) disagreed that they feel adequately prepared and supported to provide care to VI patients. This can be contrasted to the fact that 69.0% (n = 162) of respondents did not agree that they have been provided with sufficient training and guidance to provide care to VI patients. Similarly, only 10.3% (n = 24) believed that the pharmacy team(s) they work with are adequately trained to care for VI patients. Most pharmacists would welcome training to both help identify VI patients (90.6%, n = 213) and to guide management of VI patients (92.3%, n = 216). When asked specifically if they would like updated guidance regarding how to care for VI patients, the response was extremely positive with 91.5% (n = 215), stating yes. A significantly higher proportion of female respondents would be interested in more updated guidance compared to male pharmacists (female 96.0% n = 144, male 84.3% n = 70; *p* < 0.05).

**Table 2:** Agreement with statements relating to preparedness, support and training provided

Statement	Strongly agree % (n)	Agree % (n)	Neutral % (n)	Disagree % (n)	Strongly disagree % (n)
I feel adequately prepared and supported to provide care to VI patients if necessary	2.6% (6)	31.2% (73)	33.8% (79)	30.3% (71)	2.1% (5)
I have been provided with sufficient training and guidance to provide care to VI patients	2.1% (5)	8.1% (19)	20.9% (49)	54.5% (128)	14.5% (34)
The pharmacy team(s) I work with are adequately trained to care for VI patients	0.9% (2)	9.4% (22)	33.2% (78)	46.0% (108)	10.6% (25)
I would welcome training to help me identify VI patients	37.4% (88)	53.2% (125)	6.4% (15)	2.6% (6)	0.4% (1)
I would like training to guide me in the management of VI patients	36.3% (85)	56% (131)	5.1% (12)	2.1% (5)	0.4% (1)

### Priorities when dispensing medications to VI patients

**Fig. 1:**
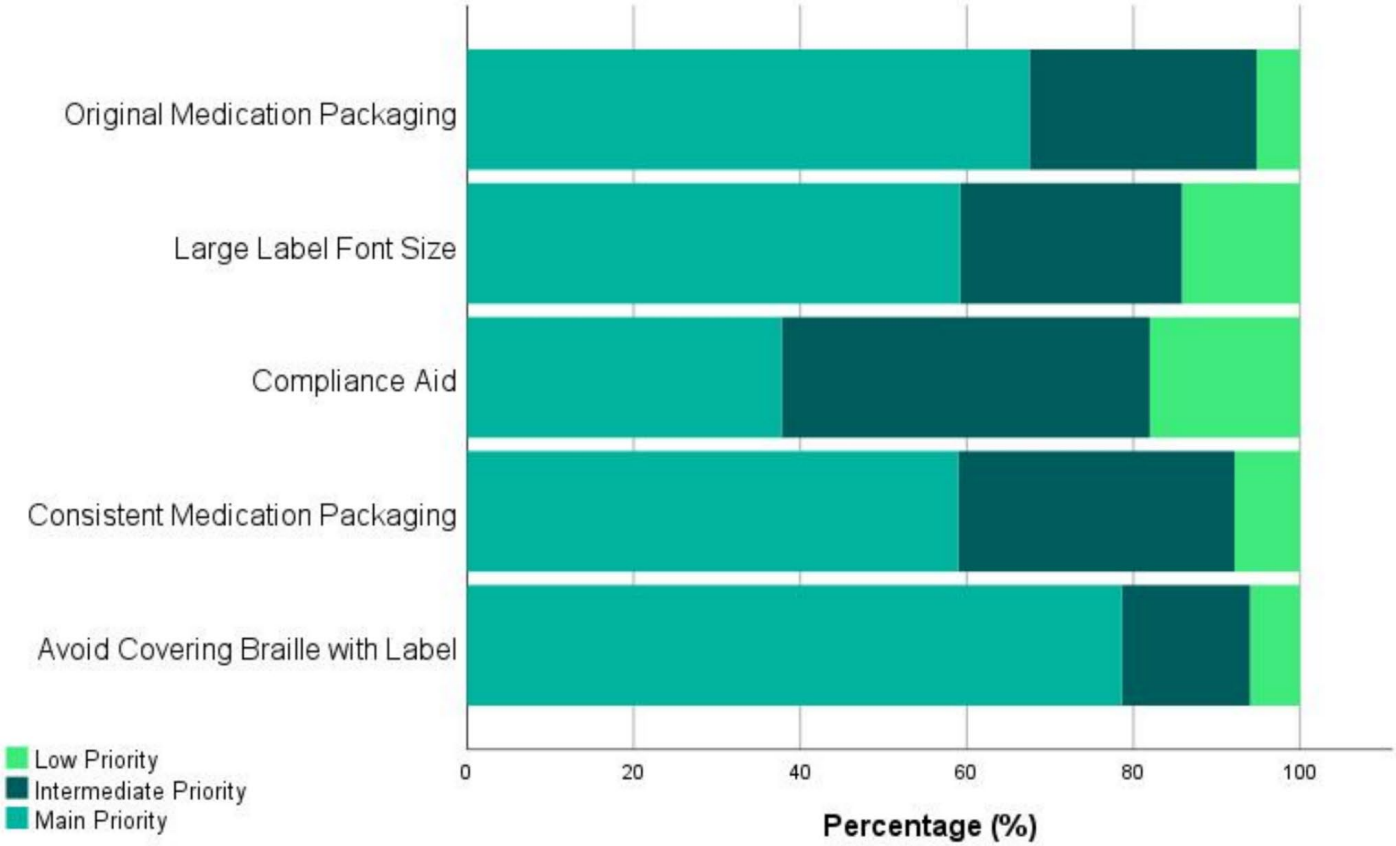
Main, intermediate and low priorities for pharmacists when dispensing medications for VI patients

As shown in Fig. 1 avoid covering braille with label was rated as a main priority by the highest number of pharmacists (78.6% n = 184), followed by providing medication in original packaging. The lowest number of respondents rated providing medication in a compliance aid as a main priority 37.8% (n = 88), but this was an intermediate priority for 44.2% of pharmacists (n = 103).

### Perceived barriers to care of VI patients

Pharmacist reported current barriers to care of VI patients are shown in Table 3. Patients not disclosing their VI, insufficient training on how to tailor care for VI patients and busy working conditions were reported as strong barriers by 40.3% (n = 94), 38.6% (n = 90) and 34.0% (n = 80) of respondents respectively. The physical pharmacy layout was reported not to be a barrier by 22.3% (n = 52).

**Table 3:** Pharmacists perceived barriers to care of VI patients

Factor	Strong barrier % (n)	Moderate barrier % (n)	Weak barrier % (n)	Not a barrier % (n)
Busy Working Conditions	34% (80)	48.9% (115)	8.5% (20)	8.5% (20)
Inability to Identify / Recognise VI Patients	21.4% (50)	48.3% (113)	20.5% (48)	9.8% (23)
Patients Not Disclosing their Visual Impairment	40.3% (94)	42.9% (100)	12.9% (30)	3.9% (9)
Poor Knowledge of Needs/Preferences of Patients	22.5% (52)	53.7% (124)	20.3% (47)	3.5% (8)
Insufficient Training on How to Tailor Care for VI Patients	38.6% (90)	44.6% (104)	13.7% (32)	3.0% (7)
Communication Difficulties	12% (28)	43.2% (101)	32.9% (77)	12.0% (28)
Physical Layout of Pharmacy	10.7% (25)	26.2% (61)	40.8% (95)	22.3% (52)

### Suggestions for adaptations to practice and improved care

The results of conventional content analysis of open text suggestions on how to adapt practice for VI patients are shown in Table 4. Quotations to support the suggestions or proposed practice adaptations identified can be found in Supplementary Information 2. Recommendations related to pharmacy physical layouts, identification of VI patients, tailored medication dispensing practices, use of technology and communication methods.

**Table 4:** Pharmacist suggestions for adaptation to practice and improved care

Category	Suggestion or proposed practice adaptation
Pharmacy Layout and Premises	Accessible entrance and open plan layout Clear, wide and uncluttered walkways to pharmacy counter Avoid leaving obstacles on the floor Floor markings, good lighting and clear signage
Identification of VI Patients and Specific Needs	Patients to carry personalised visually impaired card Note regarding vision impairment added to patient medication record on dispensing software Public health promotion campaign to encourage disclosure of disability Build relationship with patients Ask patients for clear instruction on how they want medications packaged or how they differentiate medications
Dispensing of Medications	Consistent medication branding and shapes Dispense in original packaging with braille, avoid split packs or plastic bags Large label fonts Do not cover braille with dispensing label Use of compliance aids Accessible formats e.g. large font, braille
Use of Technology	Audio recording, voice notes of medication information Use of QR codes with medicines information Intelligent devices e.g. audio-assisted glucose monitor Magnifying glass available in pharmacy Text to speech software capabilities and applications
Communicating with VI Patients	Use of consultation room to minimise distractions Ask for preferred communication method Provide verbal introduction of name and pharmacy role Inform patients verbally when moving away Ensure adequate time for consultation, demonstrate patience Speak clearly and slowly Use of Teach-Back method Provide pharmacy phone number for follow-up

## Discussion

### Statement of key findings

The study highlights how, at present, according to those surveyed sufficient training and guidance has not been provided to community pharmacists and their teams on how to care for VI patients. Currently knowledge and confidence deficits exists amongst the community pharmacists surveyed on how best to adapt care for these patients even though most study participants believe this cohort are at increased risk of medication related harm. Perceived strong barriers to providing care to VI patients include patients not disclosing their VI, insufficient training and busy working conditions. It is clear, most participants surveyed would welcome updated guidance and introduction of training on this topic.

### Strengths and limitations

This study contributes data on practising pharmacists’ experiences providing care to VI patients and the challenges currently faced by staff, which remains an under researched topic. Questionnaire development considered previous studies in the general research area and the questionnaire included both qualitative and quantitative sections to facilitate in depth mixed method data analysis. It is acknowledged that this study is limited by providing p-values above or below the 0.05 level, rather than reporting specific values. The response rate was seemingly low at 5.4%, however this response rate is in line with previous studies who have used this method for study dissemination [25], while it is also noted that not all of those sent the questionnaire may be currently working in community pharmacy. Sample size calculations were not conducted. The dissemination method facilitates a wide variety of responses owing to publicity of the study to pharmacists from all parts of the country. However, it is acknowledged the study is limited to responses from one country, but results can provide context for the international reader to interpret within the international pharmacy landscape.

### Interpretation of findings

Results highlight that less experienced pharmacists reported less confidence in providing care to VI patients versus more experienced pharmacists. This suggests that on one hand confidence comes with practical community pharmacy experience but also suggests that modern pharmacy educational programmes are not placing emphasis on providing students with skills and knowledge to care for patients with disabilities. This may to be adding to a perceived lack of confidence. Exposure to disability training appears to be sparse in pharmacy education. From an international perspective, similar results have been identified, with emphasis placed on a need for improved integration of disability training into pharmacy curricula [2, 26]. Literature does reveal various methods to expose pharmacy students to disability training, including use of simulation [27] and interprofessional learning based activities [28], however it appears that widespread inclusion in curricula is still lacking. Given the results obtained, there is a clear need for inclusion of disability training for pharmacy students, newer graduates and practising pharmacists alike.

Study results highlight the need for updated guidance and training for practicing community pharmacists and their teams on how to care for VI patients. Here, pharmacists reported feeling unprepared and a lack of support, training and guidance in this area even though 70% had previously provided care or advice to VI patients. This sentiment is consistent with previous studies from countries like Belgium, Saudi Arabia, Thailand and Scotland [18, 20–22]. It is interesting to note here that responses differed regarding pharmacist perceptions of their own training versus wider pharmacy team training availability. A higher proportion of pharmacists disagreed that they themselves have been provided with enough training. However, this is likely because the currently study focused on pharmacist responses while future work could better understand wider pharmacy teams’ preparedness. In the present study, there was a resoundingly positive response to potential updated guidance and training for pharmacists and their teams to care for VI patients. Guidance initiatives for medication management and recognition of VI patients have been developed in countries such as Belgium and Australia [29–31]. However, it appears clear that creativity in means of delivery of information is needed. As identified, pharmacists are already dealing with extremely busy working conditions, thus, information needs to be provided in a digestible and accessible manner to improve uptake and therefore patient care quality. Appropriate methods to equip pharmacists with knowledge could be via development of podcasts, continuing professional development topics, online seminars, in addition to more traditional leaflets, guidance documents and more formal micro credentials or training courses for interested staff.

Appropriate training and information for both students and practicing pharmacists will help to overcome some of the barriers to caring for VI patients highlighted here. For example, inability to identify VI patients was seen to be a strong barrier. This issue has been highlighted previously as in several international studies as across different countries patients often do not disclose their visual impairment [18, 21, 32]. Information campaigns encouraging patients to disclose any relevant disabilities to pharmacy staff and recognition tips for staff could help to overcome this perceived barrier. Additionally, VI patients often receive medication information that does not need their specific needs [14]. Better guidance for pharmacists is required on how to provide medication information in an appropriate form, given the lack of knowledge identified from respondents in this study.

Here pharmacists provided recommendations on adaptions to practice for VI patients. The recommendations, in general, echoed those from both VI patients and previous studies of pharmacists [18, 20–22]. In recent times a clear emphasis has been placed on use of technology and assistive products to assist these patients with medication management [6, 30, 33]. Technology can be easy to use and transferable to community practice, however there is not a one size fits all approach and patient preference, and their technological capabilities are key. While assistive products and applications may not be suitable for all patients, communicating effectively and building professional relationships with patients is important for all VI patient interactions. Irrelevant of their individual capabilities or level of independence. Tailored communication is essential in all interactions and should be at the forefront of adaptations to practice.

### Future research

This study provides impetus for development and introduction of educational interventions in modern pharmacy undergraduate programmes to increase exposure to disability awareness and educate students on how to adapt practice for VI patients. Tailored guidance and training programmes for practising pharmacists in digestible and accessible formats should be developed using recommendations from this study and a previous qualitative study involving VI patient recommendations [14].

## Conclusion

This study revealed valuable insights into community pharmacists’ experiences, confidence and recommendations for adapting care for VI patients. Current barriers to care as well as confidence and knowledge deficits on how best to help VI patients manage medicines were identified. Lack of training and guidance for community pharmacy staff at present is clear, despite some positive examples of good dispensing practices. Most respondents are open to updated guidance and training on this topic, and opportunities exist to include disability training in undergraduate pharmacy programmes. Despite recent emphasis on use of technology and assistive products, is clear that open communication and development of personal relationships with patients remain the cornerstones to ensuring community pharmacies are inclusive environments for VI patients.

## Supplementary Information

Below is the link to the electronic supplementary material.

Supplementary file1 (DOCX 26 kb)

## Data Availability

The datasets generated during and/or analysed during the current study are available from the corresponding author on reasonable request.
