# Supplementary material for: A cross-sectional evaluation of community pharmacists’ opinions on caring for visually impaired patients: pharmacist experiences, confidence and recommendations for improved care
Source: Int J Clin Pharm. 2026 Mar 18;48(4):1424–32. doi: 10.1007/s11096-026-02120-4 (PMC13368962; doi:10.1007/s11096-026-02120-4)
Supplement: Supplementary file 1 — Supplementary file1 (DOCX 26 kb) [file 11096_2026_2120_MOESM1_ESM.docx]

**Supplementary Information**

**A Cross-Sectional Evaluation of Community Pharmacists’ Opinions on Caring for Visually Impaired Patients: Pharmacist Experiences, Confidence and Recommendations for Improved Care.**

Journal: International Journal of Clinical Pharmacy

Aoife Goodwin-Boers, Harriet Bennett-Lenane*.

School of Pharmacy, University College Cork, Cork, Ireland.

*Corresponding Author, email: hbennettlenane@ucc.ie

**Supplementary Information 1**

**Questionnaire**

1. **Which of the following best describes how you identify your gender?**

- Male
- Female
- Non-binary
- Prefer not to answer.

1. **Select the community pharmacy role that best describes you.**

- Pharmacy Owner
- Superintendent Pharmacist
- Supervising Pharmacist
- Support Pharmacist
- Locum Pharmacist
- Other(s)

1. **How many years of post-registration experience do you have in community pharmacy?**

- <1 year
- 1 – 5 years
- 6 – 10 years
- 11 – 20 years
- >20 years

1. **Which best describes the type of pharmacy you most commonly work in?**

- Small pharmacy chain (<10 pharmacies)
- Large pharmacy chain (≥10 pharmacies)
- Independent pharmacy

1. **Which best describes the location of the pharmacy you most commonly work in?**

- Rural village or small town
- Large town
- City centre or surrounds

1. **Do you believe the physical layout of the pharmacy you most commonly work in to be accessible to patients with visual impairments?**

- Yes
- No
- Unsure

1. **Do you believe visually impaired patients are at increased risk of medication-related harm?**

- Yes
- No
- Unsure

1. **Have you previously provided care or advice, in any form, to a patient with visual impairment?**

- Yes
- No

1. **How confident do you feel providing care to patients with visual impairments?**

- Not at all confident
- Slightly confident
- Moderately confident
- Very confident
- Extremely confident

1. **Have you ever asked for feedback from your visually impaired patients on how to improve the care you provide to them?**

- Yes
- No

1. **How confident would you feel providing care to patients with visual impairments, if required?**

- Not at all confident
- Slightly confident
- Moderately confident
- Very confident
- Extremely confident

1. **Please rate your agreement with these statements:**

|  | Strongly agree | Agree | Neither agree nor disagree | Disagree | Strongly disagree |
| --- | --- | --- | --- | --- | --- |
| I feel adequately prepared and supported to provide care to patients with visual impairments if necessary |  |  |  |  |  |
| I have been provided with sufficient training and guidance to provide care to patients with visual impairments |  |  |  |  |  |
| The pharmacy team(s) I work with are adequately trained to care for people with visual impairments |  |  |  |  |  |
| I would welcome training to help me identify patients with visual impairments |  |  |  |  |  |
| I would like training to guide me in the management of patients with visual impairments |  |  |  |  |  |

1. **To date, have you completed any additional training, either formal (i.e. lectures or workshops) or informal (i.e. self-learning) regarding caring for patients with visual impairments?**

- Yes
- No

1. **Please specify the type of training.**

Free Text.

1. **Would you be interested in updated guidance regarding how to care for patients with visual impairments in community pharmacies?**

- Yes
- No
- Unsure

1. **What adaptations to your practice do you/would you make if communicating or providing advice to visually impaired patients?**

Free Text.

1. **To what extent are the following a priority for you when/if dispensing a prescription only medication for a patient with visual impairment?**

|  | Main Priority | Intermediate Priority | Low Priority |
| --- | --- | --- | --- |
| Avoiding covering braille with the label |  |  |  |
| Providing medications in original packaging |  |  |  |
| Providing medications in consistent packaging |  |  |  |
| Providing a medication compliance aid e.g. blister pack |  |  |  |
| Increasing the font size on label |  |  |  |

1. **Would you know how to obtain accessible formats (e.g. braille, audio) of medicines-related information if requested?**

- Yes
- No
- Unsure.

1. **In the course of your work have you previously been required to obtain accessible formats (e.g. braille, audio) of medicines-related information?**

- Yes
- No
- Can’t remember

1. **For the factors below, please rate the extent to which you believe these are barriers to providing improved care to patients with visual impairments in community pharmacies.**

|  | Not a barrier | Weak barrier | Moderate barrier | Strong barrier |
| --- | --- | --- | --- | --- |
| Busy working conditions of community pharmacy |  |  |  |  |
| Inability to identify/recognise patients with visual impairments |  |  |  |  |
| Patients not disclosing their visual impairment |  |  |  |  |
| Poor knowledge of specific needs/preference of the patients |  |  |  |  |
| Insufficient training on how to tailor care for visually impaired patients |  |  |  |  |
| Communication difficulties |  |  |  |  |
| Physical layout of the pharmacy |  |  |  |  |

1. **Please provide any suggestions that you believe could aid pharmacists to provide improved care to visually impaired patients?**

Free Text.

**Supplementary Information 2**

**Supportive Quotations for Conventional Content Analysis of Open Test Questions relating to Suggestions for Adaptations to Practice.**

| **Category** | **Supportive Quotations** |
| --- | --- |
| **Pharmacy Layout and Premises** | *“We welcome guide-dogs and try to ensure that all walk ways through our shop are wide and uncluttered.”* ***(Pharmacist 16)***  *“People with visual impairments usually attend our pharmacy with or via relatives, friends or carers so we have feedback. We have patient (waiting area) seats just inside the door.”* ***(Pharmacist 100)***  ***“****Lay out could be a problem but very difficult to deal with. Keep floor clear of impediments.”* ***(Pharmacist 62)***  *“There would need to be better markings on the floor to guide a person to the counter. There would be signs for products in braille.”* ***(Pharmacist 199)***  *“Ensure the layout of the pharmacy is better suited to the patients.”* ***(Pharmacist 76)*** |
| **Identification of VI Patients and Specific Needs** | *“Identify the patients with an impairment, note it on their file. Ensure to discuss the impairment the patient is encountering and document same and any extra requirements which they would benefit from.”* ***(Pharmacist 91)***  *“Ask the patient what you can do to help them understand their meds/ better utilise their meds -- every patient is different in what they require”* ***(Pharmacist 225)***  *“Patients could carry a card with them which states their level of impairment and any preferences they have on how we should communicate with them.”* ***(Pharmacist 154)***  *“Alert between pharmacists and GPs highlighting visual impairment.”* ***(Pharmacist 63)***  *“Something should be done nationally to encourage patients to disclose their disability.”* ***(Pharmacist 72)*** |
| **Dispensing of Medications** | *“Offer to blister pack medications to make it easier to comply with prescription. Use larger fonts on labels and leaflets or sheets with dosage instructions.”* ***(Pharmacist 16)***  *“Braille must never be covered. Where possible to leave medication in the original packaging, not zip-loc bags.”* ***(Pharmacist 125)***  *“Where possible try to offer consistent brands of medicine that allow patient to feel difference in shape and size of tablets. Try to avoid similar size and shape tablets”* ***(Pharmacist 132)***  *“Availability of supplementary braille labels with common dosages which could be used by pharmacist to affix to dispensed medicines”* ***(Pharmacist 172)***  *“Ensuring I choose products with braille over ones without. Ensuring label does not cover braille. Using larger print on labels…”* ***(Pharmacist 189)*** |
| **Use of Technology** | *“I have provided verbal counselling that the patient has recorded on their phone to access later. I have signposted patients to online resources that they can access with text to speech software.”* ***(Pharmacist 98)***  *“Magnifying equipment available in the pharmacy and audio descriptions of patient information leaflets”* ***(Pharmacist 85)***  *“Provided a diabetic patient with a speaking blood glucose monitor.”* ***(Pharmacist 152)***  *“Maybe how to make a specific QR code with all the appropriate recordings for their medicines or a digital hub they can access.”* ***(Pharmacist 135)***  *“By providing an app on the phone which allows audio records of medication and dosing for visually impaired.”* ***(Pharmacist 227)*** |
| **Communicating with VI Patients** | *“Use consultation room to reduce distraction and outside disturbance.”* ***(Pharmacist 209)***  *“Getting to know patient personally and handing them dispensed medicine and taking the time to discuss it, any changes and make sure they understand how to take it. Provide number to contact pharmacy or pharmacist directly if there is any issue.”* ***(Pharmacist 72)***  *“Patience, patience, patience & patient first!”* ***(Pharmacist 178)***  *“More time counselling them on the correct way to use/take their medication. Asking them to share back the information I have given them so I am confident that they understand.”* ***(Pharmacist 179)***  *“Ensure their understanding of how to take medications using teach back method…”* ***(Pharmacist 2)***  *“Tell them I am talking to them in case can’t see me, give my name and that I am pharmacist and when I am moving away”* ***(Pharmacist 72)*** |
